# Assessment of Tissue Eosinophilia as a Prognosticator in Oral Epithelial Dysplasia and Oral Squamous Cell Carcinoma—An Image Analysis Study

**DOI:** 10.1155/2014/507512

**Published:** 2014-02-19

**Authors:** Megha Jain, Sowmya Kasetty, U. S. Sudheendra, Manisha Tijare, Samar Khan, Ami Desai

**Affiliations:** ^1^Department of Oral Pathology and Microbiology, Peoples Dental Academy, MIG, Block-C, Flat No. 14, PCMS Campus, Peoples Hospital, Bhopal, Madhya Pradesh 462037, India; ^2^Department of Oral Pathology and Microbiology, Peoples College of Dental Sciences and Research Center, Bhopal, Madhya Pradesh 462037, India; ^3^Department of Oral Pathology and Microbiology, Coorg Institute of Dental Sciences, Virajpet, Karnataka 571218, India; ^4^Department of Oral Pathology and Microbiology, Rishiraj Dental College, Bhopal, Madhya Pradesh 462036, India

## Abstract

Association of tissue eosinophilia with oral squamous cell carcinoma has shown variable results ranging from favourable to unfavourable or even having no influence on prognosis. Also, very few studies have been done to know the role of eosinophils in premalignancy. So the present study investigated role of eosinophilic infiltration in oral precancer and cancer and its possible use as a prognosticator. 60 histopathologically proven cases (20 cases each of metastatic and nonmetastatic oral squamous cell carcinoma and oral leukoplakia with dysplasia of various grades) were included. Congo red is used as a special stain for eosinophils. Each specimen slide was viewed under high power in 10 consecutive microscopic fields for counting of eosinophils. As a result, a significant increase in eosinophil count was found in oral carcinomas compared to dysplasia. Nonmetastatic cases showed higher counts than metastatic carcinomas. So, it is concluded that eosinophilia is a favourable histopathological prognostic factor in oral cancer. Moreover, higher eosinophil counts in carcinoma group compared to dysplasia group proved that they might have a role in stromal invasion thus suggesting that quantitative assessment of tissue eosinophilia should become a part of the routine histopathological diagnosis for oral precancer and OSCC.

## 1. Introduction

Eosinophils were first described by Wharton Jones in 1846 as “coarse granular cells” and later by Paul Ehrlich in 1880 as “eosinophils” [1]. Eosinophils are characterised by presence of abundant cytoplasm with coarse reflective granules [2] and are distinguished by their tinctorial properties showing bright red staining with acid aniline dyes [3]. Eosinophils are pleiotropic, multifunctional leucocytes and play an important role in health and disease. They are involved in initiation and propagation of diverse inflammatory responses including parasitic helminth, bacterial and viral infections, tissue injury, and allergic diseases as well as modulators of innate and adaptive immunity [4]. Extensive tissue eosinophilia has also been described in many cancers including oral squamous cell carcinoma [5].

Tumor-associated tissue eosinophilia (TATE) is defined as “eosinophilic stromal infiltration of a tumor not associated with tumor necrosis or ulceration.” It was first described by Przewoski in 1896 in carcinoma of cervix [6]. It is characterised by the presence of eosinophils as a component of peritumoral and intratumoral inflammatory infiltrate [7, 8]. TATE in malignancies is associated with different sites such as nasopharynx [6, 9], larynx [10, 11], esophagus [12], colon [13, 14], cervix [15], external genitalia [16], skin [17], gastrointestinal tract [18], and oral cavity [7, 8, 16, 19–27].

Eosinophils are hypothesized to have direct tumoricidal activity associated with release of cytotoxic proteins and also act indirectly by enhancing the permeability into tumor cells facilitating penetration of tumor-killing cytokines. Additionally, the eosinophils may promote tumor angiogenesis by the production of several angiogenic factors. These cells also contain preformed matrix metalloproteinases (MMP) such as MMP-9 as well as their inhibitors TIMP-1 and TIMP-2 indicating that they can also modulate extracellular matrix formation. A highly potent and selective eosinophil chemoattractant, eotaxin, mainly derived from tumor-associated eosinophils is partly involved in eosinophils chemotaxis to the tumour [5]. Likewise, mast cells secrete histamine and eosinophil chemoattractant factor (ECF) which further attract eosinophils in tissues [27].

Correlation of tissue eosinophilia with prognosis has shown variable results in oral squamous cell carcinoma. It has been related to a favourable and [7, 10, 19, 20] to an unfavourable [15, 21] prognosis or even having no influence on patient outcome [6, 24].

Very few studies have been conducted to know the role of eosinophils in premalignancy. Although, certain studies have compared eosinophil counts between in situ neoplastic lesions and invasive neoplastic lesions with higher counts in latter thus suggesting that elevated eosinophil counts are a histopathological marker associated with stromal invasion [11, 22].

Although intact eosinophils can be easily identified in tissue sections that are stained with hematoxylin and eosin staining, sometimes these granulocytes assume an uncommon morphology making their recognition difficult in routinely stained sections. In such situations, special technique like autofluorescence or immunohistochemistry is needed to detect the presence of intact or degranulating eosinophils particularly in tumors [3, 28]. Moreover special stains like Congo red and carbol chromotrope also proved to be a valuable diagnostic tool for detection of eosinophils because of their unique property to bind with eosinophils [26, 27].

So, the study was aimed to elucidate the role of eosinophilic infiltration in oral precancer and cancer and its possible use as a prognosticator in oral squamous cell carcinoma using Congo red stain.

## 2. Materials and Methods

After obtaining ethical clearance, 60 intraoral histopathologically proven cases (20 cases each of metastatic and nonmetastatic oral squamous cell carcinoma (OSCC) and oral leukoplakia with dysplasia of various grades) were included in the study (Table 1).

The haematoxylin and eosin stained sections of all cases were observed under microscope. To reduce the interobserver variability, stained sections were graded for dysplasia using Burkhart and Maerkar [29] grading system by four separate examiners and OSCC using Broders grading [30]. The grading was decided when at least three observers agreed on the same grade. For counting of eosinophils, 5 *μ*m formalin fixed paraffin embedded tissue sections were obtained and stained with Congo red stain.

### 2.1. Congo Red Staining Procedure

Firstly, sections were deparaffinized, hydrated through graded alcohols to water, and then placed in 1% Congo red solution for 8 minutes followed by washing in water. Then differentiation was done in 2.5% KOH solution by dipping once. Sections were counterstained with hematoxylin for 8 minutes then washed under running tap water. Differentiation was done in 1% acid alcohol by dipping once. Lastly, the sections were dehydrated through alcohol and cleared in xylene. Finally, sections were mounted with DPX.

### 2.2. Counting of Eosinophils and Acquiring Digital Images

**Figure 1:**
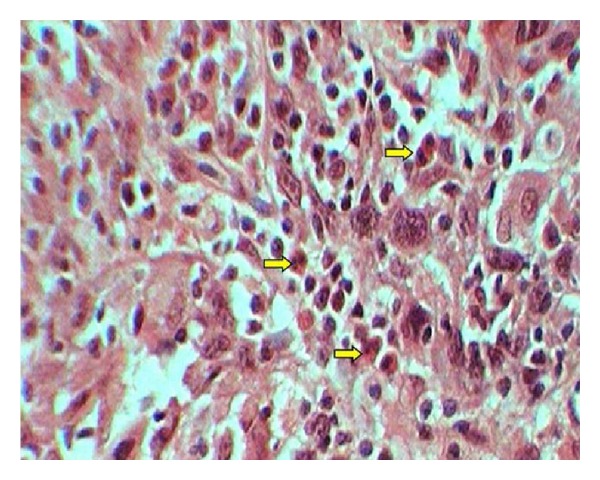
Showing eosinophils in Congo red stained sections of metastatic OSCC.

**Figure 2:**
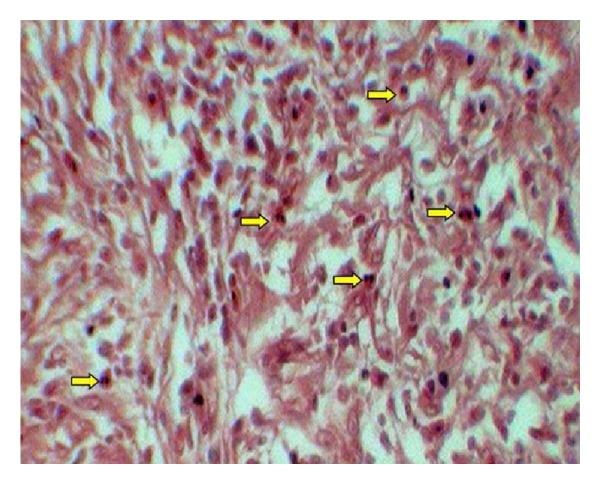
Showing eosinophils in Congo red stained sections of nonmetastatic OSCC.

**Figure 3:**
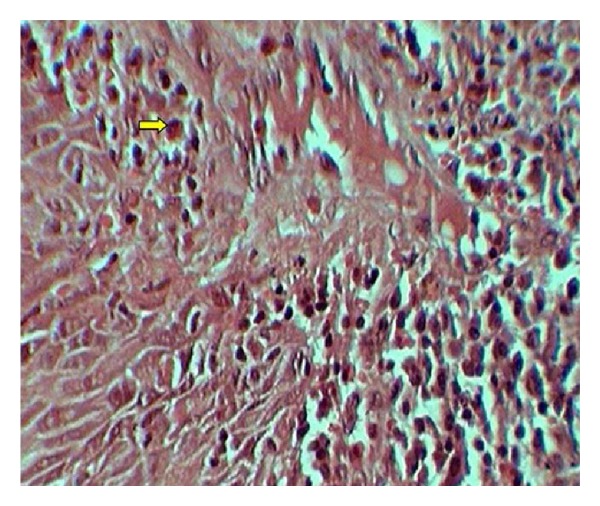
Showing eosinophils in Congo red stained sections of oral epithelial dysplasia.

Each specimen was viewed under high power (40x) microscopic field for counting of eosinophils. High power field diameter of microscope used was 0.5 mm. In case of OSCC, invasive front region was chosen for eosinophils estimation. The eosinophils were counted in 10 consecutive high power fields (hpf) and recorded as eosinophils/10 hpf [13]. Areas of tumor necrosis and degenerated muscle tissue areas have been excluded. Figures 1, 2, and 3 show the associated eosinophils in metastatic and nonmetastatic OSCC and oral epithelial dysplasia, respectively.

### 2.3. Statistical Analysis

Data was transferred to the excel sheet followed by statistical analysis using unpaired *t* test and analysis of variance (one way ANOVA) using SPSS software. A value of *P* < 0.05 was considered statistically significant.

## 3. Results

### 3.1. Assessment of Eosinophils in Each Study Group

Comparison of eosinophil counts among different grades of dysplasia assessed by one way ANOVA did not revealed any statistical significance (Table 2).

Comparison of eosinophil count between OSCC and dysplasia assessed by unpaired *t* test showed significant increase in eosinophil count in OSCC compared to dysplasia (Table 3).

Comparison of eosinophil counts between metastatic (group I) and nonmetastatic (group II) OSCC assessed by unpaired *t* test showed significantly raised eosinophil counts in nonmetastatic compared to metastatic OSCC (Table 4).

Among metastatic group, WDSCC has significantly higher eosinophilic counts compared to MDSCC, while in case of nonmetastatic group, the difference was statistically insignificant assessed by unpaired *t* test (Table 5).

Among overall WDSCC and MDSCC, nonmetastatic group has higher eosinophil count compared to metastatic assessed by unpaired *t* test (Table 6).

## 4. Discussion

The development of invasive cancer is not simply a result of genetic alterations within the tumor cell itself but is also associated with profound changes in host stromal, endothelial, and inflammatory/immune cells [8]. The peritumoral and intratumoral inflammatory infiltrates found in tumors have been considered as the host's immune response to the neoplasia [7, 8]. The initial recruitment and activation of eosinophils towards the tumour microenvironment is a complex process that is mediated by inflammatory cytokines and chemokines and is principally related to Th2 response. IL-4 and IL-13 are potent inducers of eotaxin chemokines that can explain the eosinophilia associated with Th2 responses. Eosinophil activation involves chemotactic factors like histamine and eosinophilic chemotactic factor A in mast cells, neutrophil peptides, eosinophil stimulator and promoter substances in lymphocytes, C5a complement, and eotaxin [2]. Although eosinophils are commonly encountered in human cancer, their functional role in malignancy remains an ambiguity. The literature demonstrates a tendency to consider TATE as a favourable prognostic factor in head and neck squamous cell carcinoma (HNSCC), but TATE has also been related to a poorer prognosis or even to no influence on patients' outcome reflecting that this issue is still a matter of controversy [7, 8]. Therefore, the present study was attempted to investigate the role of TATE in oral precancer and OSCC and whether it can be used as a predictive marker for OSCC.

In the present study, we have compared mean eosinophil count among mild, moderate, and severe dysplasia group but the difference was found to be statistically insignificant. Till now, none of the studies have considered grades of dysplasia as a parameter for counting of eosinophils and this needs further researches.

In our study, mean eosinophil count in OSCC group was found to be significantly higher than dysplasia group, suggesting that they might have a role in stromal invasion. The finding is in support with Alrawi et al. [22] who demonstrated elevated eosinophilic counts in invasive squamous cell carcinoma compared to noninvasive tumors of head and neck region. Similarly, findings by Falconieri et al. [23] suggested that SCC with eosinophil rich reactive inflammatory infiltrate is consistently associated with stromal invasion. Oliviera et al. [8] found that intense eosinophilia was strongly associated with advanced staged T3/T4. Said et al. [11] also reported elevated eosinophil counts in invasive laryngeal neoplasm compared to noninvasive (preinvasive) neoplastic lesions suggesting it as a morphologic feature associated with tumor invasion.

But the finding is in contrast to study by Moezzi et al. [13] who concluded that in the spectrum of colonic neoplasms, stromal eosinophilia is most prominent in adenomas and seems to decrease with progression through the adenoma-carcinoma sequence. Kiziltaş et al. [14] also reported that that intensity of TE declined with increasing malignant potential of colonic epithelial neoplasms and may be used as diagnostic indicator.

In this study, mean eosinophil count in nonmetastatic OSCC group was found to be significantly higher than metastatic group indicating that eosinophils have a good prognostic role in OSCC. This finding is in accordance with Goldsmith et al. [19, 20] who found that TE was significantly associated with favourable outcome in SCC of head and neck and concluded that high grade TATE was the most influential among various histopathological variables affecting clinical outcome. Dorta et al. [7] found that intense tissue eosinophilia was associated with 72% of 5-year disease-free cumulative survival whereas only 32% and 44% were associated with absent/mild and moderate tissue eosinophilia, respectively. Falconieri et al. [23] also confirmed that eosinophil rich SCC, although association with metastatic involvement of cervical lymph node seems to persue a less aggressive behaviour if compared with ordinary SCC. Debta et al. [26] found that increase infiltration of eosinophils and mast cells in OSCC were associated with favourable prognosis. Thompson et al. [10] also reported that TATE is associated with good term prognosis for laryngeal carcinoma. Ohashi et al. [12] found that cases of esophageal squamous cell carcinoma without lymph node metastasis had a significantly larger number of tumor-associated eosinophilia than those without lymph node metastasis.

But the studies in contrast to our result include those by Horiuchi et al. [21] whose findings revealed that the degree of eosinophil infiltrate and expression of HLA-DR antigen on tumor cell were significant prognostic factors associated with unfavourable prognosis in case of well differentiated OSCC. Alrawi et al. [22] also demonstrated that patients with high eosinophil indices had a statistically significant lower survival than those with lower eosinophil indices. Alkhabuli and High [31] found insignificant correlation between eosinophil density (ED) and survival and lymph node metastasis. Oliviera et al. [8] reported equivalent 5-year and 10-year overall survival and disease-free survival rates for both OSCC with intense and absent/mild tissue eosinophilia. Likewise, Tadbir et al. [24] concluded that TATE has no correlation with prognostic parameter in OSCC. Leighton et al. [6] assessed the presence of TATE in nasopharyngeal carcinoma and found that TATE was not significantly associated with local recurrence, distant metastasis, and survival.

As per the study, mean eosinophil count of WDSCC and MDSCC of no-metastatic group was significantly higher than metastatic group. However when mean eosinophil count within the group was compared, in metastatic group, WDSCC had significantly higher counts over MDSCC, but in nonmetastatic group, difference was found to be insignificant. So far, none of the studies have considered such categories of parameters but with regard to overall tumor differentiation few studies do exist. Iwasaki et al. [18] found a significant association between low degree of tumor cell differentiation and strong eosinophilic infiltration suggesting that some special histologic type of carcinoma may preferentially attract eosinophil into the lesion. Alkhabuli and High [31] did not find any correlation between eosinophil density and SCC differentiation in cases of SCC of tongue. Similarly, Tadbir et al. [24] failed to report any significant correlation between TATE and tumor differentiation in patients of OSCC. Also, Rahrotaban et al. [25] demonstrated no significant correlation between TATE and histopathologic grading, but it was lower in poorly differentiated group than in others in cases of HNSCC.

In conclusion, the present study assessed role of tissue eosinophilia as a prognosticator in oral precancer and OSCC (metastatic and nonmetastatic) and found tissue eosinophilia as a favourable histopathological prognostic factor in OSCC. In addition, we have found higher eosinophil counts in OSCC group compared to dysplasia group justifying that they might have a role in stromal invasion. So, the present study recommends that quantitative assessment of eosinophils should become a part of the routine histopathological diagnosis for oral precancer and OSCC. Also, one should be more cautious if higher eosinophil counts are evident in dysplastic lesions that prompt a thorough evaluation for invasiveness.

## Figures and Tables

**Table 1:** Showing distribution of study sample.

Study groups	Description of groups	Number of cases
Group I	Metastatic OSCC	**20** Well-differentiated squamous cell carcinoma (WDSCC) = 05 Moderately differentiated squamous cell carcinoma (MDSCC) = 15
Group II	Nonmetastatic OSCC	**20** WDSCC = 12 MDSCC = 08
Group III	Dysplasia	**20** Mild dys = 12 Moderate dys = 04 Severe dys = 04

**Bold values** indicate total no. of cases that is 20 (in each category).

**Table 2:** Depicting comparison of eosinophil counts among different grades of dysplasia.

Group	Grades	Mean	SD	*P* value	Result
Dysplasia *n* = 20	Mild (*n* = 12)	2.117	1.369	0.652	Nonsignificant
	Moderate (*n* = 04)	2.850	1.085		
	Severe (*n* = 04)	2.325	1.537		

**Table 3:** Depicting comparison of eosinophil counts between OSCC (group I and II) and dysplasia.

Group	Mean	SD	*P* value	Result
OSCC (Group I and II) *n* = 40	6.565	3.350	<0.0001	Significant

**Table 4:** Depicting comparison of eosinophil counts between metastatic (group I) and nonmetastatic (group II) OSCC.

Group	Mean	SD	*P* value	Result
Group I (metastatic) *n* = 20	4.275	2.038	<0.0001	Significant
Group II (nonmetastatic) *n* = 20	8.855	2.800		

**Table 5:** Depicting comparison of eosinophil counts between different histological grades of metastatic (group I) and nonmetastatic OSCC (group II).

Group	Grades	Mean	SD	*P* value	Result
Group I (metastatic) *n* = 20	WDSCC (*n* = 5)	5.900	2.891	0.0355	Significant
	MDSCC (*n* = 15)	3.733	1.412		
Group II (nonmetastatic) *n* = 20	WDSCC (*n* = 12)	8.208	2.239	0.2145	Non-significant
	MDSCC (*n* = 8)	9.825	3.407		

**Table 6:** Depicting comparison of eosinophil counts between the same histological grades of metastatic (group I) and nonmetastatic OSCC (group II).

Group	Grades	Mean	SD	*P* value	Result
Group I (metastatic) *n* = 20	WDSCC (*n* = 5)	5.900	2.891	0.0075	Significant
Group II (nonmetastatic) *n* = 20	WDSCC (*n* = 12)	8.208	2.239		
Group I (metastatic) *n* = 20	MDSCC (*n* = 15)	3.733	1.412	<0.0001	Significant
Group II (nonmetastatic) *n* = 20	MDSCC (*n* = 8)	9.825	3.407		
